# Radiation quality matters: morphological and biochemical responses of *Brassica rapa* microgreens to X-rays, C-ions, and Fe-ions

**DOI:** 10.1007/s00425-025-04835-6

**Published:** 2025-10-10

**Authors:** Sara De Francesco, Chiara Amitrano, Ermenegilda Vitale, Giulia Costanzo, Walter Tinganelli, Mariagabriella Pugliese, Cecilia Arrichiello, Paolo Muto, Marco Durante, Stefania De Pascale, Carmen Arena, Veronica De Micco

**Affiliations:** 1 Department of Agricultural Sciences, University of Naples Federico II, Portici, 80055 Naples, Italy; 2 Department of Biology, University of Naples Federico II, 80126 Naples, Italy; 3 Biophysics Department, GSI Helmholtzzentrum Für Schwerionenforschung GmbH, Planckstraße 1, 64291 Darmstadt, Germany; 4 Department of Physics “E. Pancini”, University of Naples Federico II, 80126 Naples, Italy; 5 Radiotherapy Unit, Istituto Nazionale Tumori—IRCCS—Fondazione G. Pascale, 80131 Naples, Italy

**Keywords:** Extreme environments, Ionizing radiation, Leaf traits, Phytochemical countermeasures, Radiobiology, Stress response

## Abstract

**Main Conclusion:**

Radiation type and dose distinctly modulate microgreens development, revealing trait-specific thresholds where X-rays induce hormesis, carbon ions delay differentiation, and iron ions enhance biochemical balance with moderate anatomical disruption.

**Abstract:**

As space exploration progresses and controlled-environment agriculture becomes increasingly relevant under extreme conditions, understanding how ionizing radiation affects plant development is crucial. Ionizing radiation poses a major constraint in space cultivation systems, also playing a role in terrestrial stress scenarios. Despite growing interest in radiation biology, few studies have systematically compared plant responses to different radiation types with distinct linear energy transfer (LET). In this study, seeds of *Brassica rapa* L. were exposed to increasing doses of X-rays (low-LET), carbon ions, and iron ions (high-LET). Seed germination, morpho-anatomical, and biochemical traits of plants were assessed up to the microgreens stage. Plant responses were both dose- and radiation-specific. Specifically, X-rays triggered a hormetic response at low doses (1 Gy), with a decline in several analyzed traits at higher doses. Carbon ions increased leaf expansion but reduced the content of pigments, proteins, and the structural investment, suggesting a delayed tissue differentiation and low-cost acclimation mechanism under stress. Iron ions promoted a coordinated upregulation of biochemical defenses and moderate anatomical changes. Overall, radiation quality induced distinct acclimation strategies in *B. rapa*, influencing the balance between growth, structural integrity, and defense mechanisms, highlighting its notable radioresistance. Moreover, identifying trait-specific thresholds and response patterns suggests that different radiation types could be selectively applied to modulate specific functions (e.g., biomass or antioxidants promotion, anatomical adjustments) based on desired outcomes. These findings provide valuable insights into how different ionizing radiation types impact plant responses, addressing a critical gap in space-oriented research and guiding strategies to optimize plant growth in extraterrestrial environments.

**Supplementary Information:**

The online version contains supplementary material available at 10.1007/s00425-025-04835-6.

## Introduction

Plants, as sessile organisms, have evolved complex strategies to cope with various environmental stressors, including temperature fluctuations, excess or insufficient light, drought, flooding, and salinity (Lamalakshmi Devi et al. 2017; Raza et al. 2020; Dos Santos et al. 2022). These stressors can trigger complex morpho-physiological and biochemical responses aimed at maintaining homeostasis and ensuring species survival (Nadarajah 2020).

In a futuristic vision of space colonization, plants are expected to encounter environmental conditions reminiscent of ancient Earth (i.e., before land colonization), such as altered gravity perception (e.g., microgravity and milligravity *versus* buoyancy in aquatic plants, which altered the gravity perception) (McGinley and Weis 2009), and increased exposure to solar radiation (e.g., cosmic or ionizing radiation *versus* high solar irradiation due to a thinner atmosphere providing less protection) (Graham 1993). Ionizing radiation comprises X-/gamma-rays and high-energy charged particles capable of penetrating biological tissues, inducing molecular damage, oxidative stress, DNA strand breaks, and alterations in cellular homeostasis (De Micco et al. 2011; Arena et al. 2014a; Reisz et al. 2014). Different radiation types are characterized by different linear energy transfer (LET), a parameter that profoundly influences the biological outcome of radiation exposure. Low-LET (including X- and gamma-rays) is more sparsely ionizing than high-LET (including heavy ions) (Durante and Cucinotta 2011; De Micco et al. 2022; Russ et al. 2022). Both low- and high-LET radiation can cause direct damage due to direct action of radiation on a critical target molecule, such as DNA or proteins, and indirect damage due to the action of reactive oxygen species (ROS) or reactive nitrogen species (RNS) from water radiolysis and oxygen reaction with endogenous nitric oxide. Direct effects prevail after exposure to high-LET radiation, while indirect effects account for approximately two-thirds of the damage induced by low-LET radiation (Bayens et al. 2023).

Understanding plant responses to different radiation types is critical for enabling the stepwise implementation of manned space exploration missions (from Low Earth Orbit, LEO, up to Mars, passing through the Moon). The International Space Exploration Coordination Group (ISECG) has identified knowledge gaps that need to be filled to enable manned exploration. Within the key areas of “Life Support and Habitability” and “Crew Health and Performance,” the integration of astronauts’ diet with *in-situ* produced food is included (ISECG 2024). Indeed, manned missions beyond Low Earth Orbit (LEO) will require the development of Bioregenerative Life Support Systems, in which plants not only contribute to water and oxygen regeneration, waste recycling, and food production, but also serve as psychological support for the crew by helping to mitigate the stress associated with prolonged isolation and confinement (Poulet et al. 2016; Wheeler 2017; De Pascale et al. 2021; De Micco et al. 2023a; Cockell et al. 2024; ESA 2024).

Evaluating ionizing radiation effects on plants is also relevant for terrestrial radiation biology, particularly in the context of contamination following nuclear disasters, and for agricultural applications like crop improvement (e.g., mutation breeding) (Gupta and Walther 2025; Neupane et al. 2025).

Although plants exhibit greater tolerance to ionizing radiation than mammalian cells, the underlying morpho-physiological and biochemical pathways underpinning such a tolerance remain poorly understood, especially under the mixed radiation fields expected during space missions (De Micco et al. 2011; Gudkov et al. 2019; Geras’kin 2024; Volkova et al. 2022).

Ionizing radiation-induced effects in plants have so far proven to be highly species-, cultivar-, radiation- and dose-specific, as well as influenced by the interaction with other environmental and cultivation factors such as gravity and light quality (De Micco et al. 2023b). This is particularly true in the early stages of plant development, as recently demonstrated in a comparative screening of four microgreens species exposed to heavy ions: distinct morphological and pigment-related responses were observed across genotypes under identical radiation treatments and growth conditions (Amitrano et al. 2024). These findings, along with previous research, illustrate how radiation type and dose can lead to contrasting outcomes even across closely related species or genotypes. For instance, 10 Gy titanium ions altered flavonoid profiles in *Beta vulgaris* without affecting germination (Vitale et al. 2022), whereas 20 Gy X-rays boosted antioxidant capacity in *Brassica rapa* microgreens (De Francesco et al. 2023). Similarly, *Solanum lycopersicum* seeds irradiated with 25 Gy Ca-ions exhibited reduced seedling height but increased leaf carotenoids (Arena et al. 2019a). *Dolichos melanophthalmus* tolerated 10 Gy C-ions with minimal growth penalty yet marked anatomical changes (De Micco et al. 2021a).

To date, and to the best of our knowledge, no studies have systematically assessed the responses of a single food crop species exposed to multiple types of ionizing radiation with distinct LET in the context of space-oriented research. This represents a critical knowledge gap and highlights the need for more comprehensive research on how radiation quality modulates plant responses. In light of the above, in this study, we exposed dry seeds of *Brassica rapa* L. subsp. *sylvestris* var. *esculenta* to increasing doses of X-rays (0, 0.3, 1, 10, 20, and 30 Gy), high-energy carbon ions (C-ions at 0, 0.3, 1, 10, 20, and 25 Gy) and high-energy iron ions (Fe-ions at 0, 0, 0.3, 1, 10, 20, and 25 Gy), using ground-based irradiation facilities. We hypothesized that morphological, anatomical, and biochemical traits would vary as a function of radiation type and dose, at least differentiating between X-ray and heavy-ion responses, leading to beneficial, harmful, or neutral effects in *B. rapa* microgreens. Obtained data would help evaluate the potential use of this species as a nutritionally valuable crop in controlled environments and space-based cultivation systems.

## Materials and methods

### Experimental design and irradiation procedure

Seeds of *Brassica rapa* L. subsp. *sylvestris* var. *esculenta* were obtained from a local supplier (Bioseme s.c.a.r.l., Piombino, LI, Italy). Irradiation was performed on dry seeds using three types of ionizing radiation: X-rays, carbon ions (C-ions) and iron ions (Fe-ions).

The X-ray exposure was conducted at the National Cancer Institute IRCCS Fondazione G. Pascale (Naples, Italy), using a Synergy linear accelerator (Elekta) commonly employed for radiotherapy. The irradiation was performed at doses of 0.3, 1, 10, 20, and 30 Gy, using 6 MV photons delivered at 2 Gy/min. The 3D conformal radiotherapy (3D-CRT) technique was applied, with samples exposed to two opposing fields, each measuring 20 × 20 cm^2^ at the isocenter. For each dose, the dry seeds were placed on dry filter paper and leaned between two Perspex blocks (2.5 and 5 cm thick).

The heavy ions exposure was carried out at the SIS18 accelerator of the GSI Helmholtzzentrum für Schwerionenforschung (Darmstadt, Germany). Dry seeds were exposed to ^12^C-ions (240 MeV/n) or ^56^Fe-ions (1 GeV/n) at doses of 0.3, 1, 10, 20, and 25 Gy. Dosimetry procedures are detailed in Luoni et al. (2020). For comparison, the corresponding linear energy transfer (LET) in water was approximately 0.2 keV/mm for X-rays, 15 keV/mm for C-ions, and 147 keV/mm for Fe-ions, thus covering a full spectrum from low to high-LET radiation.

During all irradiation treatments, a corresponding non-irradiated control (referred to throughout the manuscript as 0 Gy) was included to provide baseline comparisons. Control seeds were always transported and handled under the same conditions as the irradiated samples, ensuring consistent environmental exposure throughout the experimental procedures.

### Microgreens cultivation

After irradiation, seeds were transferred to the laboratory and sown in pots (diameter: 7.5 cm; height: 4 cm) filled with standard gardening soil. For each irradiation type and dose, four replicates of 400 seeds were sown. Although irradiation was performed at different facilities and times, during travelling, preparation for irradiation and irradiation procedures, the dry seeds were handled under the same conditions and kept at 20 ± 5 °C. All subsequent cultivation steps were carried out under identical conditions using the same equipment, substrate, environmental conditions, and cultivation protocol. Specifically, the same growth chamber was used for all experiments, with pots incubated under controlled conditions (24 ± 2 °C, 70 ± 10% RH) for 48 h for germination. Thereafter, seedlings were exposed to white LED light at 200 ± 20 μmol photons m⁻^2^ s⁻^1^ (PPFD) under a 12 h photoperiod. Fourteen days after sowing (14 DAS), microgreens were harvested by cutting them above the substrate level.

### Morphological assessments

At harvest, the establishment percentage was calculated as the proportion of seedlings developed into microgreens with two expanded leaves, as reported in De Francesco et al. (2023). Ten microgreens per treatment were used to measure hypocotyl length and leaf area, the latter quantified by using ImageJ (U.S. National Institutes of Health, Bethesda, USA). Five microgreens per treatment were collected and immediately fixed in the F.A.A. fixation solution (5 mL of 40% formaldehyde/5 mL of glacial acetic acid/90 mL of 50% ethanol, by vol.), for microscopy analyses. For biochemical analyses, 10 g of microgreens per treatment were harvested and immediately frozen at ₋ 20 °C. Remaining materials were used to determine the fresh and dry biomass of the whole canopy. The dry biomass was obtained after oven-drying the samples at 60 °C until constant weight. Biomass was quantified per unit area and reported as kg m⁻^2^ for fresh weight (FW) and g m⁻^2^ for dry weight (DW).

### Anatomical analyses

Leaf lamina subsamples (5 × 6 mm) were dehydrated in graded ethanol up to 95%, and embedded in an acrylic resin (JB4®, Polysciences, Eppelheim, Germany). Cross-Sects. (5 μm thick) were obtained with a rotary microtome (pfm Rotary 3004 M, Bio-Optica, Milano, Italy), stained with 0.5% Toluidine Blue O in water (in volume) (Feder and O’ Brien 1968), mounted in mineral oil, and observed under a transmitted light microscope (BX 51, Olympus, Hamburg, Germany). Digital images were acquired with a camera (Olympus EP50) and functional anatomical parameters analyzed with CellSens 2.3 software (Olympus, Tokyo, Japan). The following anatomical traits were measured (Fig. S1): total lamina thickness, adaxial and abaxial epidermis thickness, palisade and spongy parenchyma thickness, percentage of intercellular spaces in the spongy parenchyma, and stomatal frequency (adaxial and abaxial) quantified along linear transects.

### Biochemical analyses

Antioxidant capacity was assessed via the FRAP (Ferric Reducing Antioxidant Power) assay, following George et al. (2004), with modifications from Costanzo et al. (2020). Powdered microgreens (0.250 g) were extracted in 60:40 methanol:water (v/v), centrifuged (18,000 g, 15 min, 4 °C), and incubated with FRAP reagents for 1 h in the dark. Absorbance was read at 593 nm using a UV–VIS Cary 100 spectrophotometer (Agilent Technologies, Palo Alto, CA, USA). Antioxidant capacity was calculated using a Trolox standard curve and expressed as μmol Trolox equivalents g⁻^1^ FW.

Chlorophylls and carotenoids were quantified following Lichtenthaler (1987). Powder (0.200 g) was extracted in ice-cold 100% acetone, centrifuged (3,200 g, 5 min), and absorbance was measured at 470, 645, and 662 nm.

Polyphenol content was determined as in Vitale et al. (2021). Samples (0.200 g) were extracted in 80% methanol at 4 °C, centrifuged (18,000 g, 5 min), mixed with 10% Folin–Ciocalteu reagent, followed by 700 mM Na₂CO₃. Absorbance was measured at 765 nm, and results expressed as mg gallic acid equivalents g⁻^1^ FW.

Protein content was determined in 0.200 g powdered samples, using the Bradford assay (Bradford 1976), with absorbance at 595 nm, and expressed as μg BSA equivalents g⁻^1^ FW.

Ascorbic acid (AsA) content was assessed using the Ascorbic Acid Assay Kit (MAK074, Sigma-Aldrich) according to Arena et al. (2019b). Frozen samples (10 mg) were homogenized in cold AsA buffer, centrifuged (13,000 g, 10 min, 4 °C), and mixed with assay buffer. Absorbance at 570 nm was used to quantify AsA concentration (ng mL⁻^1^) via a standard curve.

### Dose–response modelling

To identify threshold responses to ionizing radiation and compare trait sensitivity across radiation types, non-linear dose–response analyses were performed on selected morphological, anatomical, and biochemical traits, separately for each radiation type (X-rays, C-ions, Fe-ions). Specifically, dry weight (DW), lamina thickness, and antioxidant capacity were analyzed as representative indicators of growth, structure, and oxidative status, respectively. These traits were selected as robust, integrative proxies of leaf development, particularly suitable for early growth stages such as microgreens.

For each parameter and radiation type, a four-parameter log-logistic model (LL.4) was fitted:

$$f\left( x \right)\, = {{c\, + \,\left( {d\, - \,c} \right) \, } \mathord{\left/ {\vphantom {{c\, + \,\left( {d\, - \,c} \right) \, } {\left[ {1\, + \,\exp \left( {b\, \times \,\left( {\ln \left( x \right)\, - \,\ln \left( e \right)} \right)} \right)} \right]}}} \right. \kern-0pt} {\left[ {1\, + \,\exp \left( {b\, \times \,\left( {\ln \left( x \right)\, - \,\ln \left( e \right)} \right)} \right)} \right]}}$$

Where *b* is the slope, *c* and *d* represent the lower and upper asymptotes, and *e* corresponds to the dose producing a 50% response (EC50). Fitting was performed in *R* (package *‘drc’*) and Python (scipy.optimize) and evaluated based on the coefficient of determination (*R*^2^) and Akaike Information Criterion (AIC).

In parallel, the No-observed effect level (NOEL), which indicates the highest dose with no statistically significant difference from the control (*P* > 0.05), was identified via one-way ANOVA and Tukey’s HSD test (*α* = 0.05), using the 0 Gy control as reference. Curve plots and threshold parameters were extracted independently for each radiation–trait combination.

### Statistical analyses

Data were analyzed using the SPSS statistical package (SPSS Inc., Chicago, IL, USA), separately for each radiation type, as irradiation experiments were conducted at different times due to facility availability. Each irradiation treatment included its own control sample, transported and handled together with the irradiated seeds. Within each radiation type, data were analyzed through one-way analysis of variance (ANOVA) with dose as a factor. The SNK (Student–Newman–Keuls) multiple comparison test (*P* ≤ 0.05) was applied as post-hoc. To check for normality, the Kolmogorov–Smirnov test was performed. Pearson’s linear correlations (*P* ≤ 0.05) were calculated between anatomical traits. However, for an overall integration of morphological, anatomical, and biochemical data, multivariate analyses were performed. Principal component analysis (PCA) was conducted using PAST3 software, and Pearson correlation matrices were used to explore trait interrelationships and dose-dependent coordination across functional levels.

## Results

### Morphological analyses

Irradiation with increasing doses of X-rays and C-ions did not significantly alter the establishment percentage compared to the control group (Supplementary Fig. S2). For X-rays, values ranged between 68 ± 11% (10 Gy) and 77 ± 0.59% (0 Gy—control), while for C-ions, they varied between 69 ± 3.9% (0.3 Gy) and 82 ± 3.2% (1 Gy). In contrast, Fe-ions exposure led to a decreasing trend, with significantly lower values at 20 Gy (65 ± 1.12%) and 10 Gy (62 ± 2.61%) compared to control (80 ± 3.5%).

The three irradiation treatments differently influenced the accumulation of biomass, hypocotyl length, and leaf area (Table 1). X-ray treatment significantly influenced all analyzed parameters except leaf area. Fresh biomass significantly decreased at 0.3 Gy and increased at 1 Gy compared to the control and higher doses. This trend of variation was not maintained in the case of dry biomass, for which irradiation at all doses but 10 Gy caused a significant increase compared to the control, with a maximum value at 1 Gy. Hypocotyl length increased significantly at 1 Gy relative to the control and all other doses except 10 Gy. Leaf area was unaffected by irradiation dose.

**Table 1:** Effect of X-ray, C- and Fe-ion irradiation on fresh and dry weight of biomass (FW and DW), hypocotyl length, and leaf area of *B. rapa* microgreens from the control (0) and seedlings irradiated with increasing doses (0.3, 1, 10, 20, 30 Gy, for X-rays; 0.3, 1, 10, 20, 25 Gy, for C- and Fe- ions)

X-ray				
Dose (Gy)	FW biomass (kg/m^2^)	DW biomass (g/m^2^)	Hypocotyl length (cm)	Leaf area (cm^2^)
0	1.928 ± 0.014 b	102.6 ± 0.770 d	4.375 ± 0.250 c	1.268 ± 0.125 a
0.3	1.700 ± 0.027 c	108.5 ± 1.694 c	5.036 ± 0.222 bc	1.100 ± 0.133 a
1	2.119 ± 0.017 a	124.6 ± 0.990 a	6.148 ± 0.218 a	1.422 ± 0.154 a
10	1.908 ± 0.022 b	103.8 ± 1.221 d	5.536 ± 0.211 ab	1.529 ± 0.235 a
20	1.893 ± 0.022 b	112.6 ± 1.306 b	4.987 ± 0.250 bc	1.563 ± 0.199 a
30	1.946 ± 0.006 b	108.2 ± 0.326 c	4.873 ± 0.236 bc	0.967 ± 0.099 a
Sign	***	***	***	NS
C- ions				
0	1.076 ± 0.060 c	104.3 ± 4.727 a	5.654 ± 0.211 b	0.102 ± 0.017 b
0.3	1.695 ± 0.120 ab	114.1 ± 5.464 a	6.529 ± 0.164 a	0.198 ± 0.028 a
1	1.426 ± 0.069 b	103.6 ± 4.478 a	5.676 ± 0.235 b	0.084 ± 0.011 b
10	1.745 ± 0.129 ab	103.5 ± 3.889 a	6.435 ± 0.145 a	0.222 ± 0.043 a
20	1.890 ± 0.133 a	94.44 ± 4.153 a	6.397 ± 0.171 a	0.266 ± 0.033 a
25	1.679 ± 0.050 ab	98.42 ± 4.612 a	5.792 ± 0.156 b	0.240 ± 0.044 a
Sign	***	NS	**	**
Fe-ions				
0	2.180 ± 0.106 a	149.7 ± 7.629 a	4.656 ± 0.154 ab	0.196 ± 0.009 c
0.3	2.100 ± 0.028 a	137.4 ± 0.606 a	5.067 ± 0.125 a	0.206 ± 0.075 c
1	1.889 ± 0.056 a	133.7 ± 4.604 a	4.801 ± 0.131 ab	0.195 ± 0.067 c
10	1.824 ± 0.166 a	126.8 ± 9.624 a	4.409 ± 0.132 bc	0.254 ± 0.070 b
20	1.846 ± 0.034 a	129.6 ± 3.541 a	4.031 ± 0.176 c	0.302 ± 0.091 a
25	2.037 ± 0.086 a	140.9 ± 9.560 a	4.483 ± 0.121 bc	0.326 ± 0.093 a
Sign	NS	NS	***	***

Mean values and standard errors are shown (*n* = 4 for biomass; *n* = 10 for microgreens length and leaf area). Different letters correspond to significant differences between irradiation doses, within each radiation type, according to the Student–Newman–Keuls multiple comparison tests (*P* ≤ 0.05)

*NS* not significant

^*^, **, ***: significant at *P* < 0.05, 0.01, and 0.001, respectively

C-ion treatment significantly influenced all analyzed parameters except dry biomass. Fresh biomass increased at all doses compared to the control, with a maximum at 20 Gy. Hypocotyl length was significantly higher at doses of 0.3, 10, and 20 Gy compared to the control, 1 Gy, and 25 Gy. Leaf area was significantly increased by doses of 0.3, 10, 20, and 25 Gy compared to the control and 1 Gy.

Fe-ion treatment significantly influenced only hypocotyl length and leaf area. Hypocotyl length was significantly reduced at 20 Gy compared to the 0.3 Gy, which showed the highest value, with control and all the other doses characterized by intermediate values. Leaf area increased with dose: plants irradiated at 20 and 25 Gy had significantly larger leaves than those at 10 Gy, which in turn exceeded all other treatments and the control.

### Anatomical analyses

**Fig. 1:**
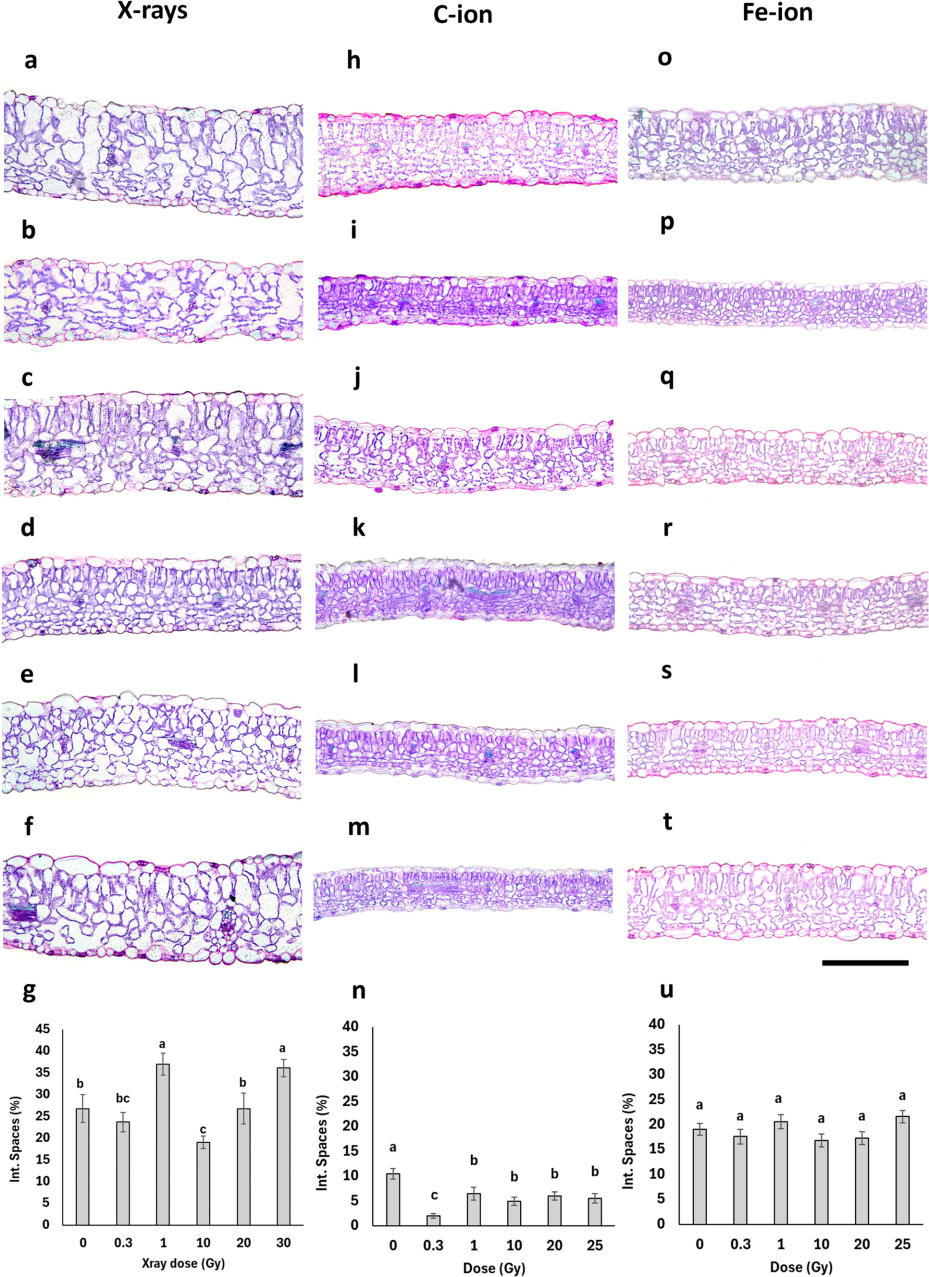
Light microscopy images of leaf lamina cross sections of *B. rapa* microgreens (**a**–**f**, **h**–**m**, **o**–**t**) grown from non-irradiated seeds (**a**, **h**, **o,** control, 0 Gy) and seeds irradiated with increasing doses of X-rays (**b**, 0.3 Gy; **c**, 1 Gy; **d**, 10 Gy; **e**, 20 Gy; **f**, 30 Gy), C-ions (**i**, 0.3 Gy; **j**, 1 Gy; **k**, 10 Gy; **l**, 20 Gy; **m**, 25 Gy), and Fe-ions (**p**, 0.3 Gy; **q**, 1 Gy; **r**, 10 Gy; **s**, 20 Gy; **t**, 25 Gy). Images are all at the same magnification. Bar = 100 μm. Quantification of intercellular space area (%) in the spongy parenchyma (**g**, **n**, **u**) for each irradiation treatment: **g**, X-rays; **n**, C-ions; and **u**, Fe-ions. Values are means ± SE (*n* = 15); different letters indicate statistically significant differences (*P* < 0.05)

**Fig. 2:**
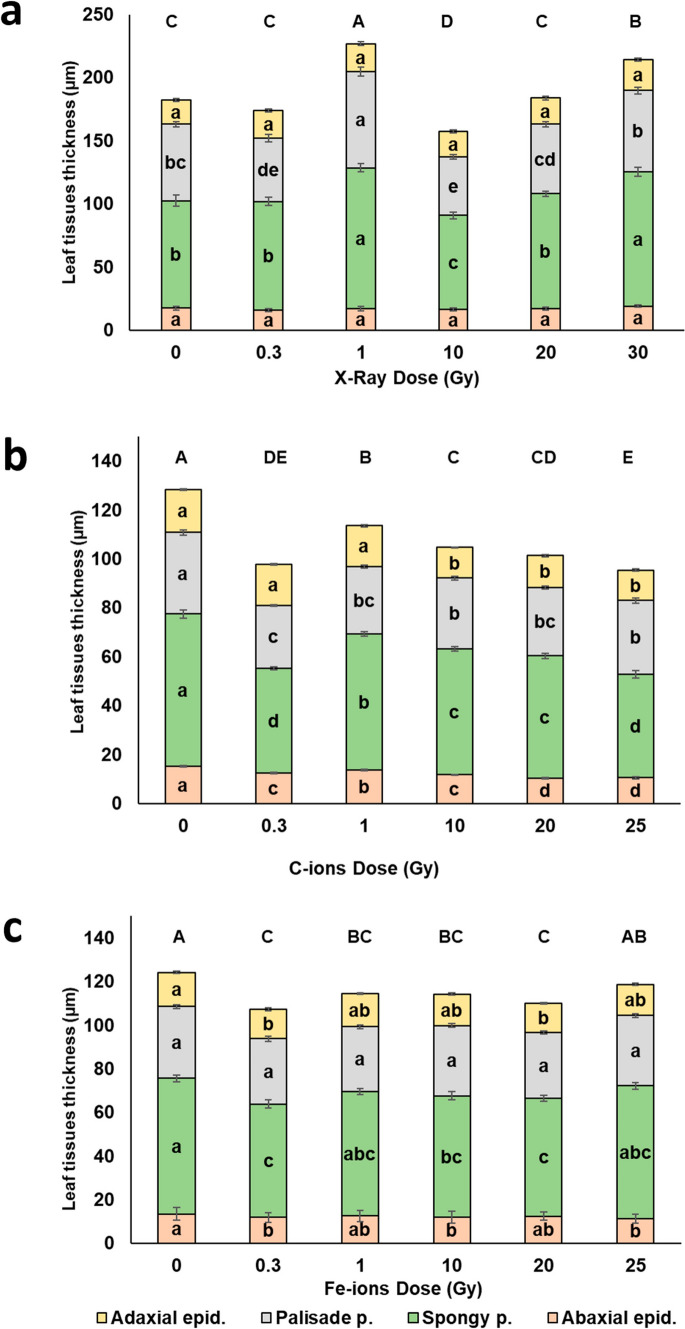
Radiation effect on the leaf anatomical traits (adaxial epidermis thickness, palisade parenchyma thickness, spongy parenchyma thickness, abaxial epidermis thickness, total leaf lamina thickness) in leaves of *B. rapa* microgreens from the control (0) and seeds irradiated with increasing doses of X-rays (**a**), C-ions (**b**), and Fe-ions (**c**). Mean values and standard errors are shown (*n* = 45). Different letters correspond to significantly different values between doses within each trait; capital letters refer to the significance of total lamina thickness

Exposure to increasing doses of X-rays, C-ions, and Fe-ions did not result in aberrations in the organization of tissues within the typical dorsiventral leaf lamina structure (Fig. 1). Thinner and more compact lamina were occasionally observed in C-ion-irradiated leaves at all doses, and in Fe-ion-irradiated leaves at 0.3 and 20 Gy (Fig. 1 i-m, p–t) compared to their respective controls, as possible signs of delayed tissue differentiation. However, from a quantitative perspective, the various irradiation treatments significantly influenced anatomical parameters (Figs. 1, 2).

**Fig. 3:**
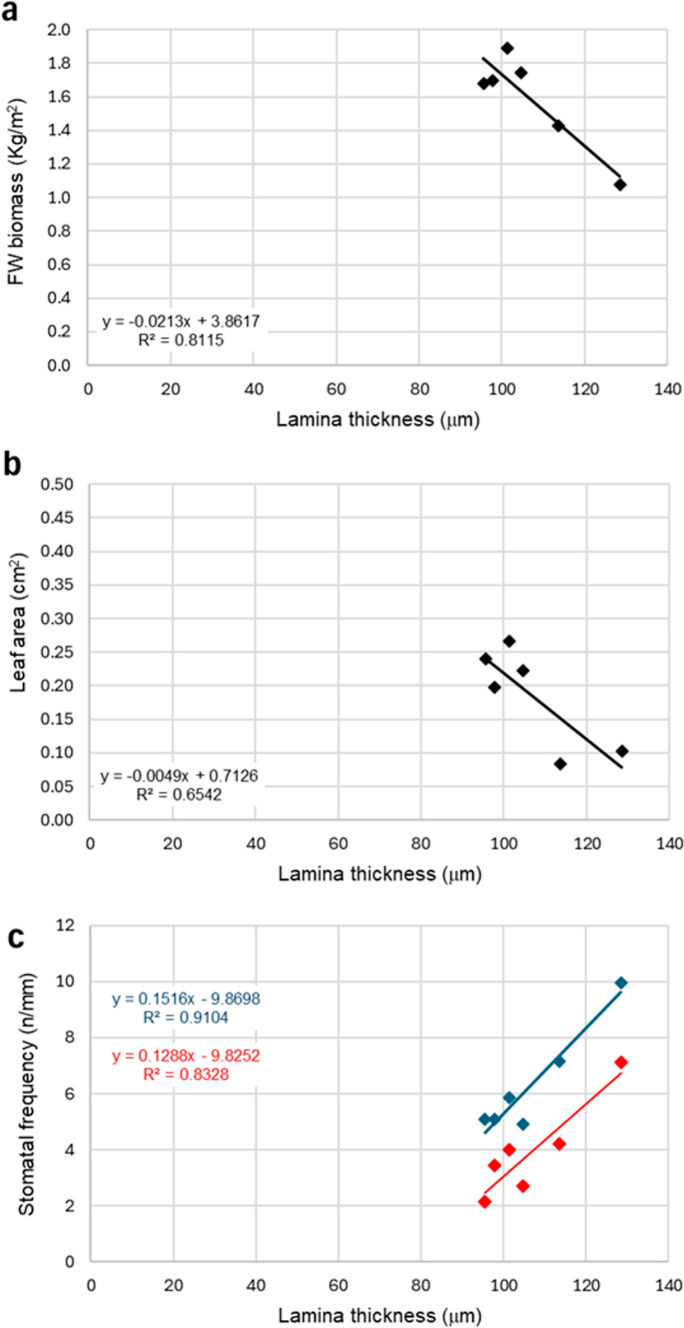
Correlations between leaf lamina thickness and FW biomass (**a**) and leaf lamina thickness and stomatal frequency on the adaxial (**b**, red) and abaxial (**b**, blue) surface in the case of microgreens from C-ions irradiated seeds. *R*^2^ values and equations of the fitting linear regressions are shown

Under X-rays (Fig. 2a), the thickness of the adaxial and abaxial epidermis remained largely unchanged across the different doses. On the contrary, the palisade parenchyma thickness showed notable variability, with a significant reduction at 0.3 and 10 Gy, as well as a significant increase at 1 Gy, compared to the control. The spongy parenchyma was significantly thinner at 10 Gy, while thicker at 1 and 30 Gy compared to the control; a similar trend was observed in the percentage of intercellular spaces, which were reduced at 10 Gy, while increased at 1 and 30 Gy compared to the control (Fig. 1g). Total leaf lamina thickness followed a similar trend to the spongy parenchyma. C-ions exposure (Fig. 2b) caused a significant decrease in epidermal thickness only at 20 and 25 Gy in adaxial epidermis, while at all doses in the case of abaxial epidermis, with minimum values at the highest doses. Both palisade and spongy parenchyma were thinner after irradiation treatments, with the lowest values observed particularly at 0.3 and 25 Gy. Consistently, total leaf thickness was significantly reduced across all doses compared to the control, with minimum values also at 0.3 and 25 Gy. Similarly, the percentage of intercellular spaces (Fig. 1n) showed a significant decrease at all irradiation doses, reaching the lowest expansion at 0.3 Gy. Concerning Fe-ion irradiation (Fig. 2c), the thickness of the adaxial epidermis significantly decreased at 0.3 and 20 Gy compared to the control. The thickness of the palisade tissue did not show significant differences across the different doses. The spongy tissue and abaxial epidermis showed the same trends of variations with significantly reduced thickness at 0.3, 10, and 25 Gy compared to the control. The total leaf thickness decreased at all doses except for 25 Gy compared to the control. In terms of intercellular spaces (Fig. 1u), no significant differences were observed between the control and irradiated samples. Leaf lamina thickness was significantly negatively correlated with fresh biomass and leaf area only for the microgreens from seeds irradiated with C-ions (Fig. 3a, b, Table S1).

Regarding stomatal frequency, no significant changes were observed under X-rays for either leaf surface (Supplementary Fig. S3a). In contrast, high-LET irradiation induced different dose-dependent variations: both leaf epidermis tissues were affected under C-ions, while the response to Fe-ions was significant only at the abaxial epidermis (Fig. S3b, c). Significant positive correlations between stomatal frequency and leaf lamina thickness were found only in the case of C-ion treatments (Supplementary Table S1).

### Biochemical analyses

The analyzed biochemical parameters were significantly influenced by radiation type and dose (Table 2). X-rays exposure significantly increased antioxidant capacity from 1 Gy, with the highest values at 30 Gy. Ascorbic acid (AsA) was significantly increased only in microgreens from 30 Gy-irradiated seeds. Polyphenol content was not significantly influenced by irradiation. Chlorophyll, carotenoid, and soluble proteins contents showed a tendency to increase with increasing doses up to 1 Gy and then to decrease again at higher doses, although the differences were not always significant.

**Table 2:** Effect of X-ray, C-ions and Fe-ions irradiation on antioxidant capacity, chlorophyll content, carotenoids, polyphenols, soluble proteins, and ascorbic acid of *B. rapa* microgreens from the control (0) and seedlings irradiated with increasing doses (0.3, 1, 10, 20, 30 Gy, for X-rays; 0.3, 1, 10, 20, 25 Gy, for C-ions and Fe-ions)

Dose (Gy)	Antioxidant capacity (μmol Trolox equivalents g^−1^ FW)	Ascorbic acid (ng mL^−1^)	Polyphenols (mg g^−1^ FW)	Chlorophyll (mg g^−1^ FW)	Carotenoids (mg g^−1^ FW)	Soluble proteins (mg BSA equivalents g^−1^ FW)
X-Rays						
0	1.652 ± 0.074 c	1.516 ± 0.086 b	0.558 ± 0.038 a	0.568 ± 0.040 bc	0.109 ± 0.008 bc	1.916 ± 0.152 ab
0.3	1.592 ± 0.062 c	1.745 ± 0.062 b	0.614 ± 0.062 a	0.706 ± 0.036 ab	0.132 ± 0.008 b	1.644 ± 0.057 b
1	2.316 ± 0.114 b	1.782 ± 0.182 b	0.586 ± 0.031a	0.855 ± 0.082 a	0.163 ± 0.015 a	2.272 ± 0.132 a
10	2.199 ± 0.063 b	1.639 ± 0.271 b	0.504 ± 0.030 a	0.624 ± 0.066 bc	0.121 ± 0.012 bc	2.012 ± 0.070 ab
20	2.326 ± 0.087 b	1.297 ± 0.088 b	0.627 ± 0.039 a	0.553 ± 0.035 bc	0.109 ± 0.006 bc	1.893 ± 0.138 ab
30	2.651 ± 0.140 a	3.775 ± 0.551 a	0.494 ± 0.024 a	0.460 ± 0.041 b	0.091 ± 0.008 c	1.603 ± 0.088 b
Sign	***	***	NS	***	***	**
C- ions						
0	3.351 ± 0.206 a	4.245 ± 0.136 a	1.166 ± 0.105 a	1.27 ± 0.041 a	0.249 ± 0.009 a	1.927 ± 0.048 a
0.3	2.349 ± 0.082 bc	3.207 ± 0.165 b	0.686 ± 0.031 bc	0.975 ± 0.037 b	0.176 ± 0.009 b	1.242 ± 0.028 b
1	2.373 ± 0.205 bc	3.262 ± 0.088 b	0.802 ± 0.058 b	0.932 ± 0.062 b	0.175 ± 0.012 b	1.023 ± 0.037 c
10	2.645 ± 0.205 b	2.892 ± 0.258 b	0.455 ± 0.048 d	1.068 ± 0.092 ab	0.163 ± 0.009 b	1.318 ± 0.029 b
20	2.139 ± 0.063 c	3.369 ± 0.099 b	0.59 ± 0.037 cd	1.076 ± 0.082 ab	0.18 ± 0.015 b	0.979 ± 0.054 c
25	2.081 ± 0.040 c	1.654 ± 0.026 c	0.515 ± 0.029 cd	1.087 ± 0.047 ab	0.189 ± 0.01 b	1.058 ± 0.044 c
Sign	***	***	***	*	***	***
Fe-ions						
0	1.305 ± 0.040 e	2.977 ± 0.077 b	0.651 ± 0.072 bc	0.984 ± 0.039 b	0.176 ± 0.007 b	0.730 ± 0.014 c
0.3	2.11 ± 0.041 d	2.765 ± 0.214 b	0.895 ± 0.060 a	0.913 ± 0.036 b	0.174 ± 0.007 b	0.793 ± 0.027 c
1	2.397 ± 0.041 c	3.598 ± 0.438 ab	0.668 ± 0.033 c	0.968 ± 0.036 b	0.177 ± 0.007 b	0.774 ± 0.014 c
10	3.759 ± 0.090 a	3.180 ± 0.176 b	0.714 ± 0.058 b	0.949 ± 0.057 b	0.173 ± 0.104 b	1.230 ± 0.087 a
20	3.730 ± 0.162 a	3.013 ± 0.147 b	0.506 ± 0.039 c	1.272 ± 0.133 a	0.248 ± 0.023 a	1.051 ± 0.031 b
25	3.107 ± 0.078 b	4.110 ± 0.284 a	0.540 ± 0.034 bc	1.034 ± 0.026 b	0.188 ± 0.008 b	1.141 ± 0.02 ab
Sign	***	**	***	**	***	***

Mean values and standard errors are shown (*n* = 8). Different letters correspond to significant different values between irradiation doses, within each radiation type, according to the Student–Newman–Keuls multiple comparison tests *P* ≤ 0.05)

*NS* not significant

^*^, **, ***: significant at *P* < 0.05, 0.01, and 0.001, respectively

In contrast, C-ion irradiation caused a general decrease in most biochemical parameters, compared to the control. The antioxidant capacity decreased the most at the highest doses (20 and 25 Gy). A similar trend was found for the AsA, which at 25 Gy was significantly lower than all the other doses, which in turn showed still higher values than the control. Chlorophylls showed a significant reduction only at doses of 0.3 and 1 Gy compared to the control. Carotenoids were significantly reduced at all irradiation doses compared to the control. Polyphenols and soluble proteins showed a decrease in their contents, not linearly, according to increasing doses.

For Fe-ion irradiation, antioxidant capacity and protein levels increased almost linearly with the dose. AsA levels were significantly higher at 25 Gy than in all the other treatments and the control, except for 1 Gy. Total polyphenol concentration showed a significant increase at 0.3 Gy compared to the control, followed by a decrease, with the lowest value observed at 20 Gy. Chlorophyll and carotenoid content significantly increased only at 20 Gy compared to all other treatments, including the control.

**Fig. 4:**
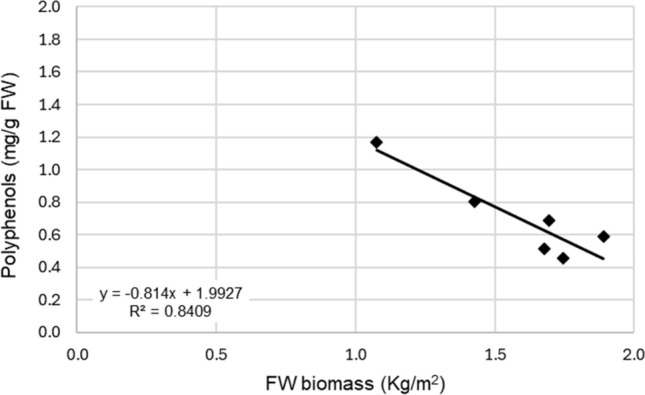
Correlation between polyphenols content and FW biomass in the case of microgreens from C-ions irradiated seeds. *R*^2^ value and equation of the fitting linear regression are shown

The polyphenol content was negatively and significantly correlated with fresh biomass only in microgreens treated with C-ions (Fig. 4, Table S1).

**Fig. 5:**
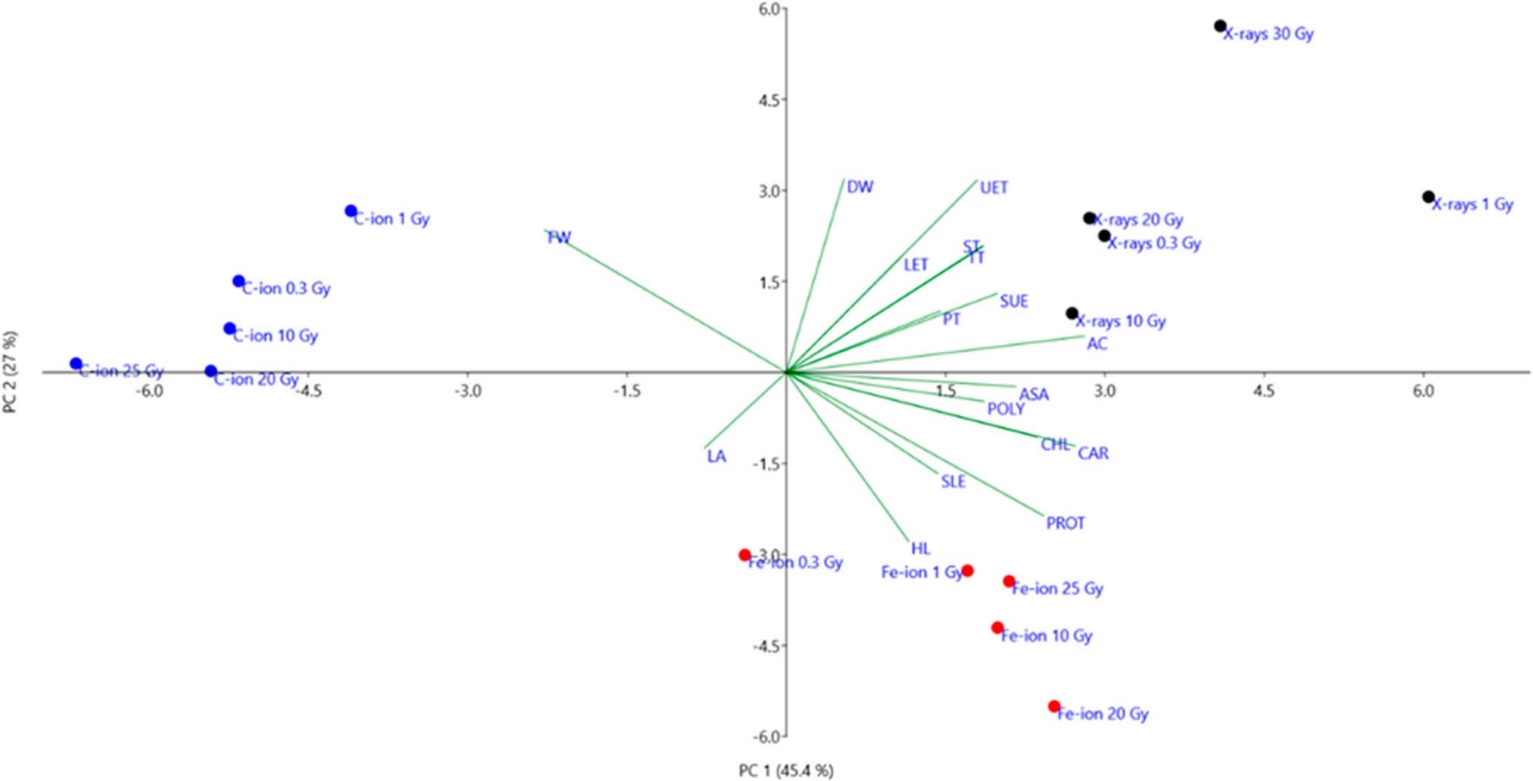
Principal component analysis (PCA) loading plot and scores of morphological (*FW* fresh weight; *DW* dry weight; *LA* leaf area; *HL* hypocotyl length) anatomical (*UET* upper epidermis thickness; *PT* palisade parenchyma thickness; *ST* spongy parenchyma thickness; *LET* lower epidermis thickness; *TT* total leaf lamina thickness; *SUE* stomata density upper epidermis; *SLE* stomata density lower epidermis) and biochemical data (*AC* antioxidant capacity; *CHL* chlorophyll; *CAR* carotenoids; *POLY* polyphenols; *PROT* soluble proteins; *ASA* ascorbic acid) in *B. rapa* microgreens from the control (0) and seedlings irradiated with increasing doses (0.3, 1, 10, 20, 30 Gy, for X-rays; 0.3, 1, 10, 20, 25 Gy) of C-ions and Fe-ions

The principal component analysis (Fig. 5) revealed that the first two components explained a cumulative 72.4% of total variance, with PC1 accounting for 45.4% and PC2 for 27%. The PCA scatterplot clearly separated the three types of radiation X-rays, C-ions and Fe-ions in the first, second and fourth quadrants, respectively. PC1 was strongly influenced by anatomical variables such as upper epidermis thickness (UET), lower epidermis thickness (LET), spongy parenchyma thickness (ST), and palisade parenchyma thickness (PT), with vectors pointing in the positive PC1 direction. PC2 was primarily associated with FW and DW. Biochemical variables, such as AsA, chlorophyll (CHL), carotenoids (CAR), polyphenols (POLY), and antioxidant capacity (AC), were associated with X-ray samples, particularly at higher doses, with vectors directed towards the bottom right quadrant.

### Dose–response modeling

Dose–response modeling revealed strong differences across radiation types and plant traits. The LL.4 model consistently outperformed other fits and provided interpretable EC50 estimates in nearly all cases (Supplementary Fig. S4). A summary of EC50 and NOEL thresholds is reported in Table 3, and curves are reported in Supplementary Fig. S4.

**Table 3:** Summary of EC50 and NOEL values extracted from log-logistic dose–response curves for three representative traits (dry weight, lamina thickness, antioxidant capacity) in *B. rapa* microgreens exposed to X-rays, carbon ions (C-ions), and iron ions (Fe-ions)

Trait	EC50 (Gy)	NOEL (Gy)
X-Rays		
Dry weight	3.33	30
Lamina thickness	0.59	30
Antioxidant capacity	0.87	30
C- ions		
Dry weight	10.23	30
Lamina thickness	0.01	25
Antioxidant capacity	0.01	30
Fe-ions		
Dry weight	0.17	30
Lamina thickness	26.33	25
Antioxidant capacity	0.81	30

Values highlight radiation-specific sensitivity patterns, with anatomical and biochemical traits responding differently across radiation types. NOEL values are model-derived and may exceed the maximum experimental dose (25/30 Gy) due to extrapolation of fitted curves

Under X-ray irradiation, trait responses followed a moderately sensitive pattern. The EC50 was 0.87 Gy for antioxidant capacity, 0.59 Gy for lamina thickness, and 3.33 Gy for dry weight, with all traits maintaining high NOEL values (30 Gy). These results indicate a hormetic or plateau-like pattern, with early responses detectable at low doses (low EC50), but no significant impairment until higher thresholds (high NOEL) were exceeded.

In contrast, exposure to carbon ions resulted in sharply divergent sensitivity profiles. Lamina thickness and antioxidant capacity showed hypersensitive responses with EC50 values approaching 0 Gy (0.00 and 0.01 Gy, respectively), while dry weight exhibited a much higher EC50 (10.23 Gy), indicating delayed growth inhibition. The NOEL was 25 Gy for lamina thickness and 30 Gy for the other traits.

Iron ion irradiation yielded an inverse trend compared to C-ions. Antioxidant capacity (EC50 = 0.81 Gy) and dry weight (EC50 = 0.17 Gy) exhibited high sensitivity, whereas lamina thickness appeared much less sensitive (EC50 = 26.33 Gy), with a lower NOEL of 25 Gy. This decoupling suggests that biochemical responses may be activated at lower doses, while anatomical traits like lamina thickness show more gradual or buffered reactions to Fe-ion exposure.

## Discussion

### Morphogenesis and structural organization

The exposure of *B. rapa* seeds to low-LET (X-rays) and high-LET (C- and Fe-ions) ionizing radiation did not impair the species’ ability to complete early development into microgreens, even at high doses (25–30 Gy). The establishment percentage remained unaffected by X-ray and C-ion exposure, while Fe-ions caused a significant reduction at 10 and 20 Gy. These results confirm the species' relative radioresistance and align with previous studies reporting variable germination responses depending on species, genotype, and radiation type (De Micco et al. 2014b, 2021b; Grasso et al. 2016; Beyaz and MacAdam 2023). Notably, contrasting trends have been observed in other Brassicaceae crops, including *Raphanus raphanistrum* L. and *Lepidium sativum* L., where carbon ion exposure showed inhibitory effects, already evident at low doses (e.g., 0.3 Gy) and persisting up to 25 Gy. In contrast, Fe-ion exposure either had no effect or even stimulated germination in the same species (Amitrano et al. 2024).

The variability in germination, lethality, and survival has been extensively documented (De Micco et al. 2011), and is not unexpected, particularly for heavy ion treatments; in the latter, direct effects dominate, and the interaction with target molecules occurs in a largely stochastic manner. Beyond germination, this high variability of responses has also been confirmed in morphological parameters and biomass accumulation. X-rays significantly stimulated biomass accumulation and hypocotyl elongation at 1 Gy, supporting the concept of radiation-induced hormesis, where mild stress promotes growth. Similar effects have been observed in *Phoenix dactylifera* L. seedlings (Al-Enezi et al. 2012) and in our previous study on *B. rapa* microgreens in which even the highest tested X-ray dose did not induce significant changes in hypocotyl length compared to the control (De Francesco et al. 2023). Similarly, hypocotyl length was not significantly reduced in *Vigna radiata* seedlings irradiated with X-rays and cultivated at specific light wavelengths (De Micco et al. 2021b).

Differently, C-ion irradiation enhanced fresh biomass and elongation at specific doses without affecting dry weight. This decoupling suggests a modulation of water status rather than carbon gain, mirroring responses observed in plants under mild drought or salinity, where turgor is maintained via osmotic adjustments without increasing dry matter (Flexas et al. 2004; Chaves et al. 2009). This observation supports our previous hypothesis that plants may acclimate to altered radiation environments by modulating stress-related signaling pathways, similar to their responses under other mild abiotic stresses on Earth (e.g., water and salt stress) (Amitrano et al. 2024).

In contrast, Fe-ion exposure suppressed hypocotyl elongation at high doses but promoted leaf lamina expansion, suggesting a dose-dependent developmental trade-off favoring horizontal expansion over vertical stem elongation. Such organ-specific shifts are consistent with acclimation strategies seen in shaded environments or under nutrient limitation, where plants prioritize leaf area expansion to optimize light capture or resource use efficiency under stress (Hermans et al. 2006; Gommers et al. 2013).

Concerning plant anatomy, X-rays irradiation caused a significant thickening of leaf lamina at 1 and 30 Gy, supporting the idea that radiation may induce anatomical plasticity to maintain photosynthetic and hydraulic efficiency under stress (He et al. 2018; Matthes et al. 2022). At 1 Gy, lamina thickening was driven by palisade parenchyma expansion and increased airspaces in the spongy parenchyma; this can likely be considered a hormetic response aimed at enhancing gas-diffusion, thus photosynthetic capacity. In contrast, thickening at 30 Gy was only driven by spongy parenchyma expansion and airspace percentage, possibly facilitating internal gas diffusion, but also suggesting early signs of structural disorganization (Amitrano et al. 2022). Similar increase in leaf lamina thickness was observed in *S. lycopersicum* after high-dose X-ray exposure, attributed to increased cell enlargement due to altered wall properties (De Micco et al. 2014b). The thickness of both adaxial and abaxial epidermal layers remained relatively stable across all X-ray doses, confirming tissue-specific responses (De Micco et al. 2014a).

Conversely, both C- and Fe-ions consistently reduced leaf lamina thickness across all doses (except Fe-25 Gy). This tendency resembled adaptations seen in shade conditions, with thin leaves characterized by large surface area (Aneja et al. 2025). However, C-ions consistently reduced lamina and mesophyll thickness across all doses, showing particularly compact tissues and reduced intercellular spaces. This structural simplification is reminiscent of shade acclimation, where reduced lamina thickness is associated with greater surface area at the expense of tissue differentiation (Aneja et al. 2025). The negative correlation between leaf area and lamina thickness in C-ion-treated plants, together with the more compact mesophyll structure, the reduced intercellular spaces, and the reduced stomata frequency, suggests a possible delayed tissue differentiation.

Fe-ions induced variable effects, with reductions in lamina thickness at low and intermediate doses but partial recovery at 25 Gy. This suggests a possible threshold-based adjustment in structural investment. Interestingly, stomatal frequency remained stable under X-rays, but decreased under C-ions and varied under Fe-ions, with C-ions showing a reduction proportionate to lamina thickness, likely a water-saving mechanism under stress (Gupta et al. 2020). Our findings align with prior work suggesting that stomatal responses to radiation are highly species- and dose-dependent (Samiyarsih et al. 2020; Albarzinji et al. 2022; De Francesco et al. 2023; Sorrentino et al. 2023).

Epidermal tissues were generally less responsive, with X-rays having no significant effect, while C- and Fe-ions caused dose-specific changes (De Micco et al. 2014a). The adaxial thickness in C-ion is reduced at high doses (10–25 Gy). The maintenance of a thickness comparable to control at low C-ion doses (notably 0.3- 1 Gy) may represent an early defense mechanism, reinforcing the outer tissue to protect against oxidative stress. Similar epidermal adaptations are known under UV-B or drought stress, where increased thickness is accompanied by cuticle deposition and water retention (Mewis et al. 2012; Zahedi et al. 2025). However, at higher doses, epidermal thickness decreased, suggesting that prolonged or intense radiation may exceed the tissue’s capacity for compensation, mirroring the concurrent reduction in mesophyll layers and stomatal density. Fe-ion irradiation, by contrast, elicited more variable epidermal responses, with dose-specific fluctuations between the adaxial and abaxial surfaces.

### Biochemical responses

X-ray and Fe-ions irradiation significantly enhanced the antioxidant capacity and ascorbic acid (AsA) content of *B. rapa* microgreens, particularly at higher doses. This trend is consistent with the activation of oxidative stress defense pathways observed in other irradiated species such as *Lactuca sativa, Cichorium endivia, Eruca sativa, and Artemisia princeps* (Fan 2005; Hwang et al. 2021; Sorrentino et al. 2023). Interestingly, polyphenol content remained unchanged across doses, with a significant increase only after 0.3 Fe-irradiation. This suggests that antioxidant enhancement induced by X-ray and Fe-ion may involve other pathways, such as increased AsA biosynthesis or activation of enzymatic antioxidants. These findings indicate an upregulation of antioxidant defenses under both low- and high-LET radiation, likely aimed at counteracting oxidative damage and stabilizing cellular membranes (Tattini et al. 2000; De Micco and Aronne 2007; Lattanzio et al. 2008; De Micco et al. 2011; Arena et al. 2014b).

From a space biology perspective, the plant's ability to boost antioxidant content has promising implications for the development of functional foods rich in dietary antioxidants (Jeong et al. 2006).

In contrast, C-ion irradiation caused a marked decline in antioxidant capacity, AsA, and total polyphenols, particularly at the highest doses. This reduction likely reflects rapid depletion of antioxidant pools in the early stages of oxidative stress, rather than suppression of biosynthesis pathways (Apel and Hirt 2004; Foyer and Noctor 2005; Sharma et al. 2012). These biochemical outcomes are consistent with anatomical responses observed under C-ion treatment, including reduced lamina and mesophyll thickness. Together, these data suggest a coordinated stress response involving both structural simplification and biochemical resource limitation, possibly driven by oxidative depletion or metabolic cost constraints (Mittler 2002; Gill and Tuteja 2010).

Under X-ray exposure, chlorophyll, carotenoids, and soluble protein levels peaked at 1 Gy before declining (returning to near-control values). This biphasic response reflects low-dose stimulation followed by stress-induced decline. The found pattern is consistent with previous observations in crops and model species, including *Arabidopsis thaliana*, *Capsicum annuum*, and *Triticum*, exposed to ionizing radiation such as γ-rays or X-rays (Gudkov et al. 2019). The concurrent anatomical thickening of the palisade parenchyma at 1 Gy supports a hormetic response coupling structure and function. The increase in protein content at the same dose likely reflects early activation of stress-responsive protein synthesis, contributing to repair and defense mechanisms (Bae et al. 2024; Yashaswini et al. 2024).

Conversely, C-ion irradiation consistently reduced pigment content, especially at high doses, and was accompanied by thinner leaf tissues, lower stomatal density, and increased leaf area. This phenotype resembles shade-acclimation strategies, in which plants minimize tissue investment while maximizing surface area, to enhance light capture under limiting or stressful conditions (Murchie and Horton 1997; Ruberti et al. 2012; Gommers et al. 2013). The simultaneous reduction in pigment levels and delay in tissue differentiation further supports the hypothesis of a low-cost adaptation mechanism under high-LET stress. In addition, the dose-dependent decline in protein content suggests systemic metabolic exhaustion (Inostroza-Blancheteau et al. 2016).

Fe-ion exposure preserved or enhanced pigment levels, particularly at 20 Gy, and maintained a more stable leaf anatomy compared to C-ions. This pigment preservation was paralleled by increased antioxidant capacity and soluble protein content, indicating a more integrated biochemical stress response, where functional adjustments are maintained. This suggests a robust and metabolically active tolerance strategy under extreme conditions (Arena et al. 2019a; Gudkov et al. 2019). This also aligns with recent transcriptomic and proteomic evidence showing that high-LET heavy ion beam (HIB) radiation induces the upregulation of antioxidant-related genes and proteins, compared to X-rays (XR), as a compensatory response to oxidative damage (Liu et al. 2023).

A cross-comparison of dose–response thresholds (EC50 and NOEL) further supports the radiation-specific nature of plant responses observed in this study. Traits linked to oxidative stress (e.g., antioxidant capacity) consistently showed the highest sensitivity across radiation types, while structural traits such as lamina thickness responded in a more variable and radiation-dependent manner. Notably, carbon ions elicited immediate effects on anatomical and biochemical traits, suggesting a low threshold of activation and potential developmental disruption at minimal doses, which would also explain delayed differentiation. X-rays induced moderate, trait-coherent responses across all metrics, while Fe-ions triggered biochemical changes with minimal structural adjustment. These findings highlight the need to consider both trait identity and radiation quality when assessing plant performance under ionizing stress, and they underscore the value of quantitative thresholds for comparing crop resilience across space-relevant conditions.

## Conclusion

This study demonstrates that radiation type and dose significantly influence growth, anatomical, and biochemical traits in *Brassica rapa* L. microgreens, shaping their acclimation strategies. They involve distinct trade-offs between growth, structural integrity, and biochemical protection, though all of them proved to be suitable in preventing detrimental outcomes. While X-rays induced a more dose-dependent hormetic response, C-ions promoted structural and metabolic suppression coupled with leaf lamina expansion. Fe-ions, in contrast, supported a coordinated stress response preserving pigments, antioxidants, and protein levels.

Importantly, the extraction of EC50 and NOEL thresholds across key traits revealed distinct sensitivity patterns depending on both trait type and radiation quality. Biochemical traits, such as antioxidant capacity, showed early and dose-dependent responses across most radiation types. At the same time, anatomical traits, particularly lamina thickness, proved to be highly responsive to specific conditions, such as carbon ion exposure, highlighting their potential as reliable structural indicators of stress. This threshold-based approach provides a quantitative framework for comparing plant sensitivity across radiation environments and strengthens trait selection strategies for space-oriented cultivation systems. These findings emphasize the importance of LET in modulating plant performance and suggest that targeted radiation treatments could be used to optimize specific traits. Identifying dose thresholds and stress markers for each radiation type is essential for selecting suitable crop candidates in future bioregenerative life support systems. Emerging concepts in induced mutagenesis and radiation breeding (Kazama et al. 2008; Guo et al. 2024) suggest that targeted use of radiation quality could become a tool for modulating growth, stress tolerance, or metabolite enrichment in crops of interest.

Despite the absence of severe abnormalities preventing microgreens morphogenesis, several variations in morphological, anatomical, and biochemical traits were observed, which were clearly radiation-type dependent, but still not linearly related to the dose. Multivariate analysis supported the hypothesis of radiation-specific responses, with principal component analysis clearly clustering the three radiation types, suggesting radiation-type-specific harmonization of traits to allow microgreens development.

## Supplementary Information

Below is the link to the electronic supplementary material.

Supplementary file1 (DOCX 1869 KB)

## Data Availability

Data will be made available upon reasonable request to the corresponding author.

## Abbreviations

AsA: Ascorbic acid
LET: Linear energy transfer
NOEL: No-observed effect level
