# Supplementary material for: Radiation quality matters: morphological and biochemical responses of Brassica rapa microgreens to X-rays, C-ions, and Fe-ions
Source: Planta. 2025 Oct 10;262(5):118. doi: 10.1007/s00425-025-04835-6 (PMC12513970; doi:10.1007/s00425-025-04835-6)
Supplement: Supplementary file 1 — Supplementary file1 (DOCX 1869 KB) [file 425_2025_4835_MOESM1_ESM.docx]

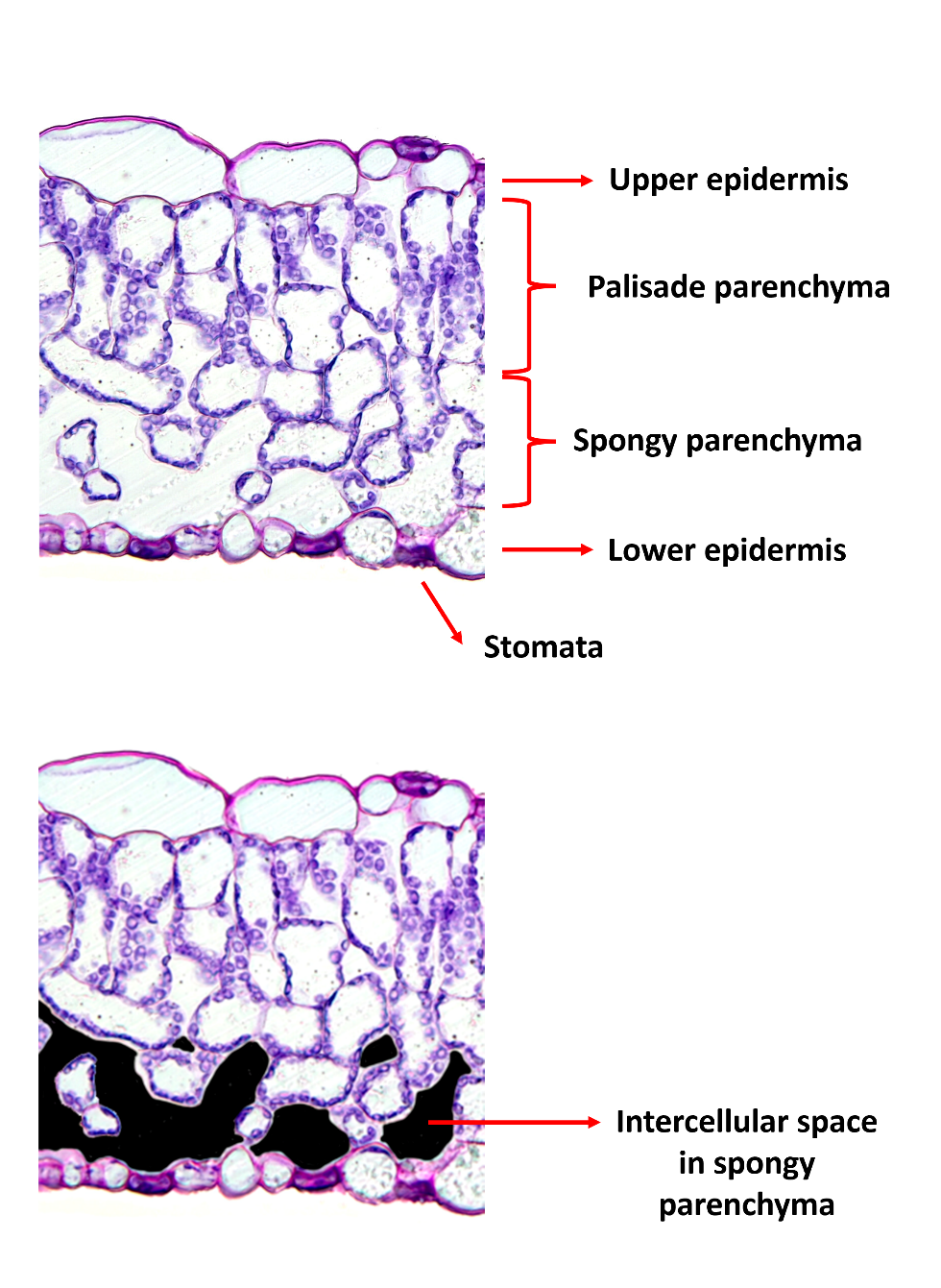
**Fig.S1.** Cross sections of a leaf of *B. rapa* L. microgreens showing the main anatomical tissues analyzed in this study.

**Fig. S2**. Radiation effect on germination percentage of *B. rapa* microgreens from the control (0) and seeds irradiated with increasing doses of X-rays (**a**), C-ins (**b**), and Fe-ions (**c**). Mean values and standard errors are shown (*n*=400). Different letters correspond to significantly different values between doses within each radiation type (*P* < 0.05).


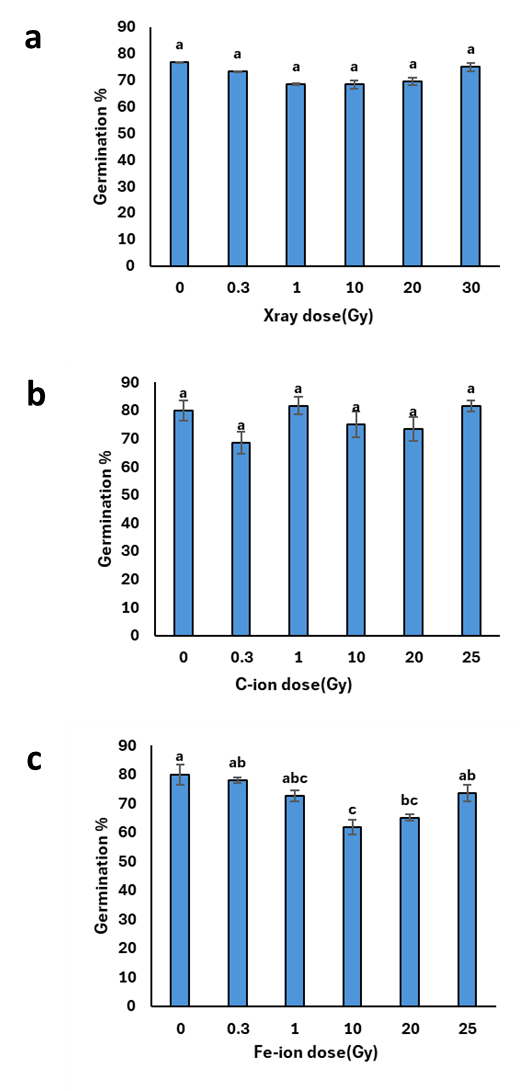


**Fig.S3**. Radiation effect on stomatal frequency of the adaxial and the abaxial surface in leaves of *B. rapa* microgreens from the control (0) and seeds irradiated with increasing doses of X-rays (**a**), C-ins (**b**), and Fe-ions (**c**). Mean values and standard errors are shown (*n*=15). Different letters correspond to significantly different values between doses, within each trait (*P* < 0.05).


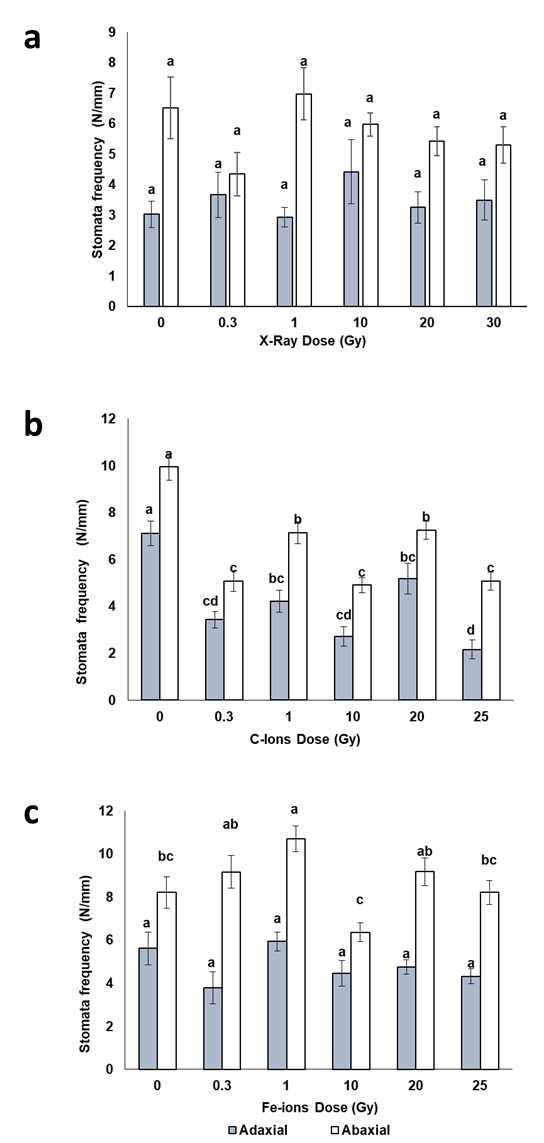


**Fig.S4**. Dose–response curves for three functional traits in *B. rapa* L. microgreens exposed to increasing doses of X-rays (**a**,**d**,**g**), C-ions (C-Ion; **b**,**e**,**h**), and Fe-ions (Fe-Ion; **c**,**f**,**i**). Traits analyzed include: dry weight (DW; **a**,**b**,**c**), antioxidant capacity (**d**,**e**,**f**), and lamina thickness (**g**,**h**,**i**). Each panel shows individual measurements and the best-fitting log-logistic curve (LL.4 model) for each radiation type, along with estimated EC50 values (dashed red lines). Curves illustrate trait- and radiation-specific response dynamics, ranging from early inhibition (C-ion) to gradual activation (X-ray and Fe-ion).


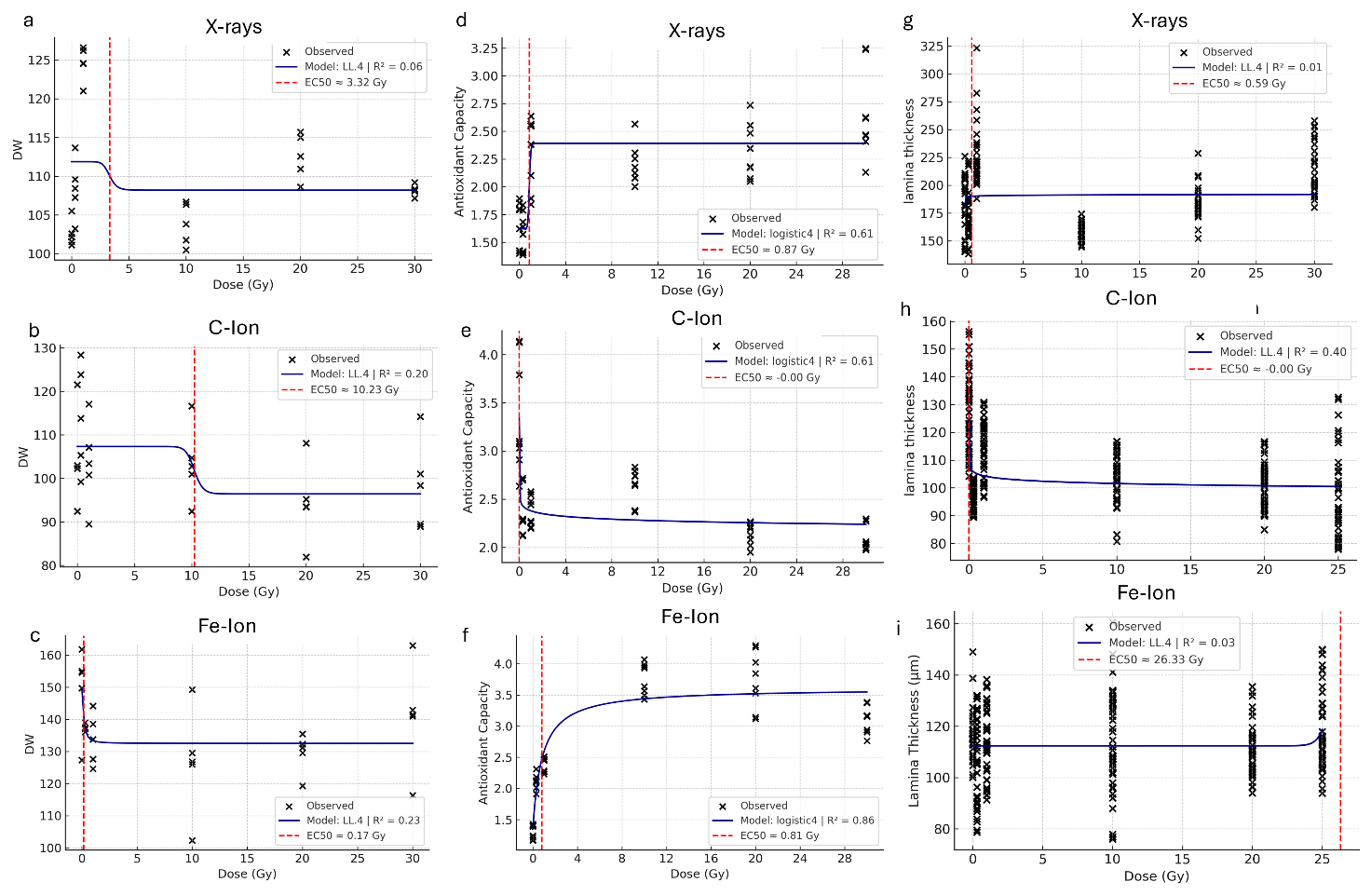


**Table S1**. Pearson correlations between selected traits for microgreens from seeds irradiated with X-rays, C- and Fe-ions. R and p values are shown.

|  |  | **Leaf lamina thickness (μm)** | | **Polyphenols content (mg/g FW)** | |
| --- | --- | --- | --- | --- | --- |
|  |  | **r** | **p** | **r** | **p** |
| FW Biomass (Kg/m^2^) | X-rays | 0.708 | 0.116 | -0.254 | 0.627 |
|  | C-ions | -0.901 | 0.014 | -0.917 | 0.010 |
|  | Fe-ions | 0.440 | 0.383 | 0.306 | 0.556 |
| Leaf area (cm^2^) | X-rays | -0.277 | 0.595 |  |  |
|  | C-ions | -0.812 | 0.050 |  |  |
|  | Fe-ions | -0.048 | 0.928 |  |  |
| Adaxial stomatal frequency (m/mm) | X-rays | 0.696 | 0.125 |  |  |
|  | C-ions | 0.913 | 0.011 |  |  |
|  | Fe-ions | 0.539 | 0.270 |  |  |
| Abaxial stomatal frequency (m/mm) | X-rays | 0.396 | 0.438 |  |  |
|  | C-ions | 0.954 | 0.003 |  |  |
|  | Fe-ions | -0.262 | 0.616 |  |  |
